# Use of geotracking in assessing point-of-sale compliance in India

**DOI:** 10.18332/tpc/156448

**Published:** 2022-12-08

**Authors:** Pradeep Aggarwal, Nandita Sharma, Mahendra Singh, Yogesh Bahurupi

**Affiliations:** 1 Department of Community and Family Medicine, All India Institute of Medical Sciences, Rishikesh, India

**Keywords:** geotracking, tobacco, smoke-free

**Dear Editor,**

Tobacco use causes approximately 5 million premature deaths worldwide each year, with the number estimated to climb to over 8 million by 2030 and directly linked to the majority of chronic lung illnesses, malignancies, and cardiovascular problems^1^. On 18 May 2003, the Indian Parliament passed the Cigarettes and Other Tobacco Products (Prohibition of Advertisement and Regulation of Trade and Commerce, Production, Supply, and Distribution) Act (hereafter referred to as COTPA)^2^. The Act covers all tobacco-containing products in any form and covers the entire country of India. Geotracking is the act of transforming raw survey data into a geomap that aids in the depiction of utility locations efficiently and correctly.

A cross-sectional survey was conducted to ascertain compliance with Section 6a of COTPA 2003, by assessing the point-of-sale where tobacco products are sold (POS) using geotracking in 6 districts of Uttarakhand namely Dehradun, Haridwar, Uttarkashi, Tehri, Udham Singh Nagar, and Champawat, over a period of 6 months. All the POS in each district were considered.

Among various POS visited, signages were displayed in 54 (4.1%). Of the displayed signage, 2.7% were at prominent places. The overall mean compliance to Section 6a (based upon the mean of major compliance indicators) was 61%. All of the 6 districts scored over 43% in compliance with major indicators of Section 6a. Tobacco products were not displayed in the majority of visited POS (Table 1).

**Table 1:** Compliance of Section 6a COTPA in points-of-sale of six districts of Uttarakhand India, 2021

*Compliance indicators*	*Champawat (n=260) n (%)*	*Dehradun (n=210) n (%)*	*Haridwar (n=211) n (%)*	*Tehri Garhwal (n=214) n (%)*	*Udham Singh Nagar (n=213) n (%)*	*Uttarkashi (n=210) n (%)*
**Mean compliance of major indicators**	180 (69.0)	110 (52.0)	109 (52.0)	128 (60.0)	144 (67.0)	131 (63.0)
**Signage displayed**	5 (1.90)	9 (4.30)	17 (8.10)	9 (4.20)	10 (4.70)	4 (1.90)
At prominent place	3 (60.0)	7 (77.8)	11 (64.70)	7 (77.80)	4 (40.0)	4 (100)
As per COTPA						
Size of signage	2 (40.0)	7 (77.8)	8 (47.10)	8 (88.90)	4 (40.0)	4 (100)
Signage in Indian	2 (40.0)	9 (100)	10 (58.80)	9 (100)	3 (30.0)	4 (100)
Size of picture	2 (40.0)	8 (88.9)	10 (58.80)	8 (88.90)	2 (20.0)	4 (100)
Size of text	1 (20.0)	8 (88.9)	9 (52.90)	8 (88.90)	2 (20.0)	4 (100)
Text	1 (20.0)	9 (100)	12 (70.60)	7 (77.80)	4 (40.0)	4 (100)

The results of the research highlighted the importance of geomapping in tobacco control. Our purpose was to determine the extent of COTPA compliance among persons and institutions, by using geotracking technology to direct law enforcement and public health organizations on where to focus enforcement and public education resources to achieve the status of ‘Smoke-free and COTPA act’ district^3^. In accordance with COTPA 2003, this study also assisted in determining people’s behavior abidance in various sectors such as public settings, government facilities, etc.^4,5^. Law enforcement and public health groups can use geomapping to make data acquisition easier and ensure compliance with COTPA^3^. The findings of this study could be used to develop an intervention strategy and ensure that COTPA is properly implemented by law enforcement agencies^3^. Thus, geotracking technology could help identify the places of concern to enable the achievement of smoke-free and COTPA-compliant countries.

## Data Availability

The data supporting this research are available from the authors on reasonable request.
